# Balancing Clinical Applicability and Scientific Depth in ML Models for MDA5-DM Prognosis: Response to ‘From machine learning to clinical practice: phenotypic clusters of anti-MDA5 antibody-positive dermatomyositis’. By Koopman, Jacob; Buhler, Katherine; Choi, May

**DOI:** 10.1093/bib/bbae295

**Published:** 2024-07-01

**Authors:** Emily McLeish, Nataliya Slater, Frank L Mastaglia, Merrilee Needham, Jerome D Coudert

**Affiliations:** Murdoch University, Centre for Molecular Medicine and Innovative Therapeutics, Murdoch, Western Australia (WA), Australia; Murdoch University, Centre for Molecular Medicine and Innovative Therapeutics, Murdoch, Western Australia (WA), Australia; Perron Institute for Neurological and Translational Science, Nedlands, WA, Australia; Murdoch University, Centre for Molecular Medicine and Innovative Therapeutics, Murdoch, Western Australia (WA), Australia; Perron Institute for Neurological and Translational Science, Nedlands, WA, Australia; University of Notre Dame Australia, School of Medicine, Fremantle, WA, Australia; Fiona Stanley Hospital, Department of Neurology, Murdoch, WA, Australia; Murdoch University, Centre for Molecular Medicine and Innovative Therapeutics, Murdoch, Western Australia (WA), Australia; Perron Institute for Neurological and Translational Science, Nedlands, WA, Australia; University of Notre Dame Australia, School of Medicine, Fremantle, WA, Australia

In their letter to the editor of Briefings in Bioinformatics Koopman *et al*. eloquently provided additional context to our recent review article on how phenotypic stratification of idiopathic inflammatory myopathies (IIMs) using machine learning (ML) models holds promising potential for developing effective diagnostic tools that will translate into clinical practice; this process is still in its early stages, with many more studies needed to replicate and validate the findings. They have expanded our argument by providing more in-depth insight specifically in the context of anti-MDA5-positive dermatomyositis (MDA5-DM).

MDA5-DM is a heterogeneous subgroup of dermatomyositis that falls under the classification of IIM. Interstitial lung disease (ILD) is a commonly associated co-morbidity, the incidence of which varies (40–100%) [1] across the literature; and among these patients, a further 30–50% will develop rapidly progressing ILD (RP-ILD) [1, 2] which carries a poor prognosis and high mortality risk. The challenge for clinicians, therefore, is predicting who will develop RP-ILD to effectively monitor, manage or treat patients.

The goal of our initial review was to provide a general overview of how ML methods have been used to help address the issue of heterogeneity within subgroups of IIMs, including MDA5-DM. We discussed how ML methods have helped evaluate the sensitivity/specificity of different diagnostic criteria and evaluate the clinical utility of specific biomarkers such as autoantibodies; we also examined how ML models can help predict co-morbidities such as malignancy or ILD. However, our discussion was more centered around utilizing ML methods for large and complex datasets such as immunophenotyping peripheral blood cell populations, and multi-omic analyses (genomic, metabolomic, etc.) [3].

Since our review was published, additional studies have used clustering methods and decision trees to stratify MDA5-DM patients as outlined by Koopman *et al*. In their letter, Koopman *et al.* highlight several important issues. Firstly, the variability in patient cohort sizes and clinical characteristics across studies leads to inconsistencies that complicate clinical interpretation. Furthermore, there are no standardized definitions or diagnostic criteria for MDA5-DM. Clinical diagnosis primarily relies on a combination of detecting MDA5 antibodies (with variable sensitivity depending on the detection methods) and IIM classification criteria for dermatomyositis or of the 2018 European Neuromuscular Centre (ENMC). As we have stated in our review:

***‘Computational methods’ reliability for biomarker discovery are limited by the accuracy of the detection methods. As such, careful consideration and validation of detection methodologies are imperative for accurate and meaningful results’***  ^***(3)***^***.***

In addition, there is no consensus on the definition for rapidly progressive (RP)-ILD, and variability in inclusion criteria between studies may lead to misclassification. It is crucial to remember that the reliability of predictive ML models is limited by the quality of the data and the clarity of the objectives provided. Our biases can be unintentionally embedded in these models, leading to predictions that may perpetuate existing prejudices. This is especially significant in healthcare, where biased predictions can impact quality of care and patient outcomes. Hence, it is essential that the data and criteria used are as unbiased, representative and consistent across studies to mitigate these risks.

Another point raised by Koopman *et al*. is that the number and variability of clinical characteristics used across studies make clinical interpretation challenging. As the use of ML models to diagnose and stratify patients is increasingly more common, the variability in inclusion criteria and measured parameters makes it challenging to identify the most appropriate for clinical decisions. Koopman *et al.* propose that the focus should be narrowed down to a few easily measurable and consistently replicated characteristics.

For instance, in their discussion they reference two studies that applied the Cox proportional hazards model and identified key characteristics including sex, disease duration, C-reactive protein and anti-Ro52 as highly predictive for RP-ILD in MDA5-DM. Although not traditionally considered an ML model the Cox proportional hazards model is the most widely accepted method for survival analysis [4]; however, it is inadequate for high-dimensional data and struggles to model nonlinearities and interaction effects [4]. Additionally, without exploring additional variables, we cannot ascertain whether the current measures are sufficiently specific. Increasing the number of variables used for modelisation may uncover more precise predictors for RP-ILD in MDA5-DM, improving diagnosis and prognosis.

There appear to be two distinct perspectives on the application of ML for predicting disease diagnosis and prognosis. This depth of knowledge has the potential to drive future studies toward the development of specialized and targeted treatments. Balancing these needs involves fostering scientific exploration and advancement while ensuring clinical applicability.

Finally, Koopman *et al.* emphasize the critical need for more rigorous replication across studies. However, stringent replication can be challenging for rare diseases like MDA5-DM, where limited cohort sizes affect the choice of model and limits its generalizability. Nevertheless, we agree that validating ML models across multiple cohorts is a crucial step to ensure reliability and applicability in clinical settings. Collaboration between research institutions and data sharing will facilitate more comprehensive and robust model validation. However, increased data also bring added complexity, which may influence the selection of the ML model that is the most suitable to handle multifaceted information.

As we try to navigate the complex heterogeneity observed among patients, it is essential to balance the scientific pursuit of understanding the deeper pathomechanisms of the disease with the need for applicable, simple and clinically relevant variables.

**Key Points**

- This manuscript discusses the potential of machine learning (ML) models to improve the phenotypic stratification of idiopathic inflammatory myopathies (IIMs), particularly focusing on anti-MDA5-positive dermatomyositis (MDA5-DM).
- MDA5-DM, a subgroup of dermatomyositis, is commonly associated with comorbidities. A significant proportion of patients develop rapidly progressing interstitial lung disease (RP-ILD). Predicting who will develop RP-ILD is critical for effective patient management.
- Unifying the variability in patient cohort sizes, clinical characteristics and lack of standardized diagnostic criteria for MDA5-DM is a major challenge that limits the reliability of ML models. There is a critical need for unbiased and consistent data across studies for rare diseases like MDA5-DM to achieve rigorous replication of ML models.
- It is crucial to integrate novel scientific insights into the complex mechanisms of disease with the development of practical and easily applicable clinical variables.
